# Traditional Chinese medicine regulating Wnt/β-catenin and OPG/RANKL/RANK pathways in osteoporosis: a narrative review based on bone coupling and pathological subtype differences

**DOI:** 10.3389/fmed.2026.1924175

**Published:** 2026-09-16

**Authors:** Shi-Lei Song, Jian-Min Li, Sheng-Yi Feng, Hai-Ming Lu

**Affiliations:** Joint Surgery Department, Yueyang Hospital of Integrated Traditional Chinese and Western Medicine, Shanghai University of Traditional Chinese Medicine, Shanghai, China

**Keywords:** bone coupling, OPG/RANKL/RANK, osteoporosis, pathological subtypes, traditional Chinese medicine, wnt/β-catenin

## Abstract

**Background:**

Osteoporosis (OP) is characterized by bone coupling imbalance—insufficient osteoblast-mediated formation and excessive osteoclast-mediated resorption. Among multiple signaling networks involved in bone remodeling, the canonical Wnt/beta-catenin pathway and the osteoprotegerin/receptor activator of nuclear factor-kappaB ligand/receptor activator of nuclear factor-kappaB axis, namely the OPG/RANKL/RANK axis, were selected because they represent two central and mutually interacting regulatory systems governing osteoblast differentiation and osteoclastogenesis. Traditional Chinese Medicine (TCM) offers multi-target potential for regulating bone homeostasis.

**Objective:**

This narrative review examines TCM interventions targeting Wnt/β-catenin and OPG/RANKL/RANK pathways in OP, emphasizing bone coupling and pathological subtype differences.

**Methods:**

Structured literature searches (PubMed, Web of Science Core Collection, CNKI, Wanfang, cqVIP; 2000–2025) with OCEBM/SYRCLE evidence grading were conducted.

**Conclusion:**

TCM suggests bidirectional bone coupling regulation through multi-target mechanisms, but evidence remains predominantly preclinical (Levels 4–5). Pharmacokinetic barriers (low oral bioavailability of icariin, berberine), safety concerns (Xianling Gubao hepatotoxicity), and lack of fracture-endpoint RCTs limit clinical translation. Future research should prioritize target-engagement biomarkers, bone-targeted delivery, and adequately powered RCTs.

## 1 Introduction

Osteoporosis (OP) represents a significant global health burden, affecting approximately 200 million people worldwide, with particularly high prevalence among postmenopausal women and elderly populations (1). The most severe clinical consequence of osteoporosis is fragility fracture, which substantially increases morbidity, mortality, and healthcare costs (2). Current anti-osteoporotic treatments include bisphosphonates, denosumab (anti-resorptive agents), teriparatide, abaloparatide, and romosozumab (bone-forming or dual-action agents), which have demonstrated efficacy in reducing fracture risk (3). However, limitations persist regarding long-term treatment sequencing, rare adverse effects, cost, compliance, and applicability in complex comorbidities and secondary osteoporosis (4).

From a modern bone biology perspective, osteoporosis is not merely “bone loss” but represents a spatiotemporal dysregulation of bone formation and resorption. The canonical Wnt/β-catenin pathway serves as the critical hub for osteoblast differentiation, while the OPG/RANKL/RANK axis represents the central system regulating osteoclast generation and maturation (5, 6). Increasing evidence suggests transcriptional and microenvironmental crosstalk between these pathways: Wnt signaling not only promotes osteogenic gene expression but also upregulates OPG and inhibits RANKL, thereby producing secondary anti-resorptive effects (7, 8). This “pro-osteogenic and anti-osteoclastic” bidirectional linkage provides the molecular foundation for understanding TCM's multi-target bone protective effects.

In Traditional Chinese Medicine (TCM), osteoporosis is categorized under “bone atrophy” and “bone withering”, with pathogenesis closely related to kidney deficiency, spleen deficiency, blood stasis, and collateral vessel malnutrition (9, 10). Recent studies indicate that TCM monomers, compound formulas, and proprietary medicines can regulate inflammation, oxidative stress, marrow adiposity, and immune microenvironment, potentially reshaping bone coupling through intervention in both Wnt/β-catenin and OPG/RANKL/RANK pathways (11–13).

Compared with recent reviews published between 2023 and 2025, which have mostly focused on individual compounds, single signaling pathways, network pharmacology predictions, or general anti-osteoporotic effects of TCM, the present review has three distinctive features. First, it integrates Wnt/beta-catenin and OPG/RANKL/RANK signaling within a unified bone-coupling framework rather than treating osteogenesis and osteoclastogenesis as independent processes. Second, it discusses pathway alterations according to pathological subtypes, including postmenopausal osteoporosis (PMOP), senile osteoporosis, glucocorticoid-induced osteoporosis (GIOP), and diabetic osteoporosis (DOP), thereby avoiding a “one mechanism fits all” interpretation. Third, it critically evaluates translational limitations, including evidence hierarchy, human pharmacokinetic uncertainty, feasibility of achieving therapeutic concentrations in bone tissue, safety risks, quality control of herbal preparations, and the current lack of fracture-endpoint clinical trials. Therefore, this review aims not only to summarize mechanisms but also to define the boundary between preclinical promise and clinical applicability.

## 2 Methods

This article is a narrative review with structured literature-search procedures. Although it was not designed as a systematic review or meta-analysis, PRISMA-inspired reporting elements were incorporated to improve transparency and reproducibility.

### 2.1 Data sources and search strategy

Literature searches were conducted in PubMed/MEDLINE, Web of Science Core Collection, CNKI, cqVIP Data, and Wanfang Data for studies published from January 2000 to December 2025. The search strategy combined controlled vocabulary and free-text terms related to osteoporosis, TCM interventions, and the two major bone-remodeling pathways. The PubMed search strategy was as follows:

(“Osteoporosis” [Mesh] OR osteoporosis OR “bone loss” OR “bone remodeling” OR “bone metabolism”) AND (“Medicine, Chinese Traditional” [Mesh] OR “Traditional Chinese Medicine” OR “Chinese herbal medicine” OR “Chinese medicine” OR “herbal formula” OR “Chinese patent medicine” OR acupuncture OR moxibustion OR icariin OR astragaloside IV OR tanshinone IIA OR salvianolic acid B OR berberine OR morroniside OR gallic acid OR “Xianling Gubao” OR “Zuogui Wan” OR “Yougui Wan”) AND (“Wnt” OR “beta-catenin” OR “LRP5” OR “LRP6” OR “SOST” OR “sclerostin” OR “DKK1” OR “OPG” OR “osteoprotegerin” OR “RANKL” OR “RANK” OR “osteoclastogenesis”).

Similar search terms were adapted for Web of Science, CNKI, cqVIP Data, and Wanfang Data. Reference lists of relevant reviews and key articles were manually screened to identify additional studies.

### 2.2 Eligibility criteria

Studies were eligible if they met one or more of the following criteria: (1) investigated TCM formulas, proprietary Chinese medicines, acupuncture/moxibustion, or isolated TCM-derived constituents in osteoporosis or bone-remodeling models; (2) reported outcomes related to Wnt/beta-catenin signaling, OPG/RANKL/RANK signaling, osteoblast differentiation, osteoclastogenesis, bone mineral density, bone microarchitecture, bone turnover markers, or fracture-related outcomes; (3) included cellular experiments, animal models, observational human studies, randomized controlled trials, systematic reviews, or meta-analyses; (4) were published in peer-reviewed journals in English or Chinese.

Exclusion criteria were: (1) conference abstracts, editorials, letters without primary data, non-peer-reviewed reports, or dissertations without accessible full text; (2) studies unrelated to osteoporosis or bone remodeling; (3) studies of non-TCM interventions unless used as mechanistic comparators; (4) duplicate publications; (5) articles lacking sufficient methodological or outcome information.

### 2.3 Study selection and evidence grading

Two authors independently screened titles and abstracts, followed by full-text assessment. Disagreements were resolved through discussion with a senior author. Extracted information included intervention type, disease subtype or model, molecular targets, bone-related outcomes, pharmacokinetic information, safety findings, and evidence level. The Oxford Centre for Evidence-Based Medicine 2011 Levels of Evidence were used to grade clinical and preclinical evidence. Cellular studies were generally considered Level 5, animal studies Level 4, small or non-definitive clinical studies Level 2b-3, and high-quality randomized controlled trials Level 1b. For animal studies, methodological quality was further considered using the SYRCLE risk-of-bias framework.

### 2.4 Literature-screening flow

The literature-screening process is summarized in Figure 1. Because this is a narrative review, the flow diagram is intended to improve transparency rather than to claim the exhaustiveness of a systematic review (Figure 1).

**Figure 1:**
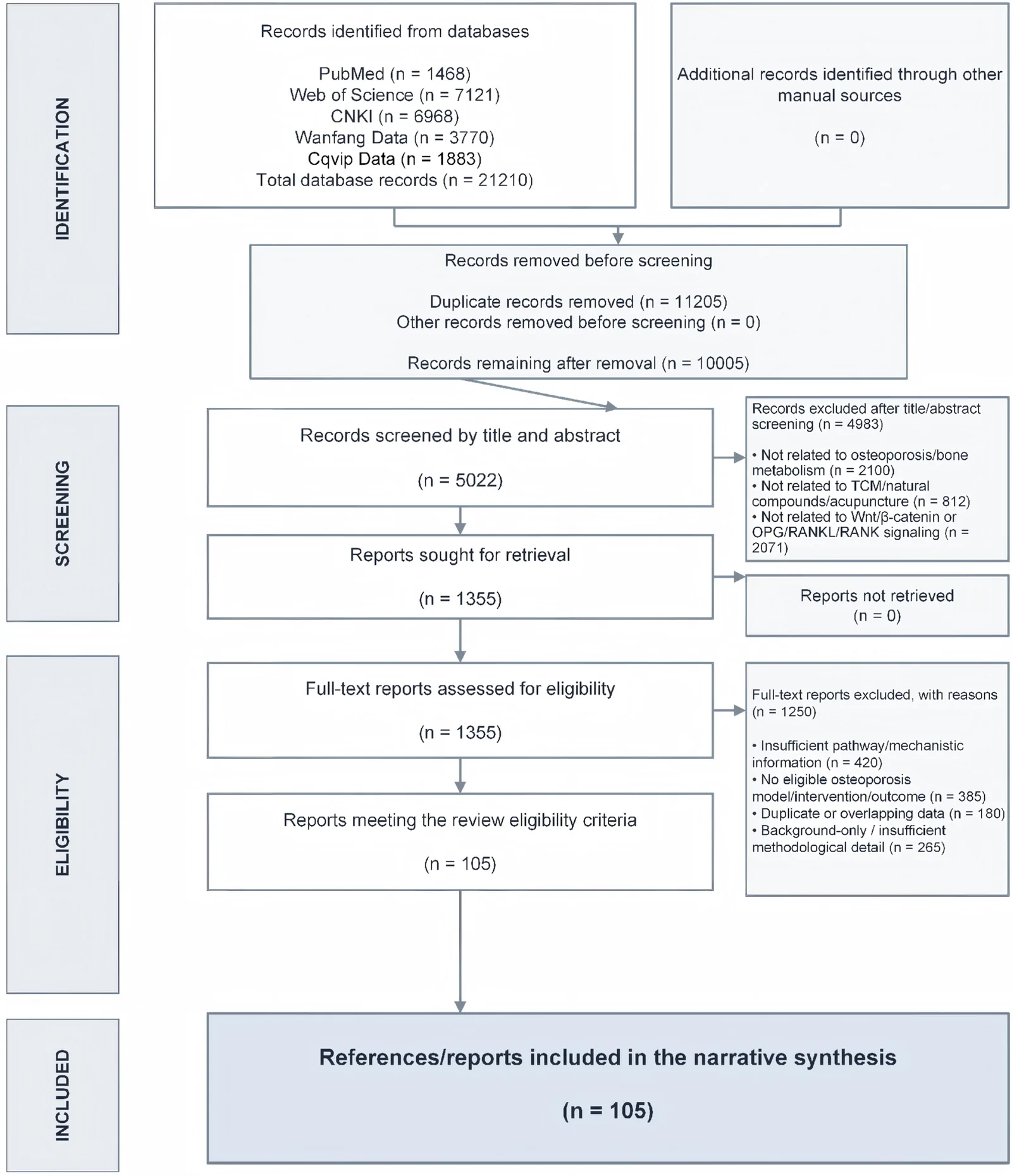
PRISMA-inspired flow diagram of literature identification, screening, eligibility assessment, and inclusion.

## 3 Bone remodeling and the central role of Wnt/β-catenin and OPG/RANKL/RANK pathways

### 3.1 Bone coupling imbalance as the common pathological core of osteoporosis

Normal bone remodeling depends on the dynamic balance between bone marrow mesenchymal stem cells (BMSCs) differentiating into osteoblasts and mononuclear/macrophage lineage cells differentiating into osteoclasts. Osteogenesis and osteoclastic activity are not independent processes but are coupled through cell-cell contact, paracrine factors, mechanical loading, and local vascular and immune microenvironments (14). When osteogenesis is insufficient, osteoclastic activity is excessive, or when both processes become imbalanced at different stages, the result is decreased bone mass, sparse trabecular structure, and reduced bone strength. Therefore, understanding osteoporosis through the lens of “bone coupling” better explains the potential advantages of TCM's approach of “simultaneous tonification and drainage, holistic microenvironment remodeling”.

### 3.2 Wnt/β-catenin signaling in osteoblast differentiation and bone formation

The canonical Wnt pathway is initiated when Wnt ligands bind to Frizzled receptors and LRP5/6 co-receptors, inhibiting the “destruction complex” composed of Axin, APC, and GSK-3β. This stabilization allows cytoplasmic β-catenin accumulation and nuclear translocation, where it associates with TCF/LEF transcription factors to upregulate osteogenic genes including Runx2, Osx, ALP, and OCN (15, 16). This pathway not only determines the osteogenic differentiation fate of BMSCs but also influences the functional status of mature osteoblasts and osteocytes.

Sclerostin (SOST) and Dickkopf-1 (DKK1) represent the most important endogenous inhibitors of Wnt signaling. Their overexpression typically indicates suppressed osteogenesis and constitutes a key event in pathological states such as glucocorticoid-induced osteoporosis (17). Mutations in LRP5 causing high bone mass phenotypes have validated the pathway's critical role in bone homeostasis (18).

### 3.3 OPG/RANKL/RANK axis in osteoclastogenesis and bone resorption

RANKL is primarily produced by osteoblasts, osteocytes, and certain immune cells. It binds to RANK on osteoclast precursor cells, recruiting TRAF6 and activating NF-κB, MAPK, and Ca²⁺/calcineurin signaling cascades, ultimately inducing c-Fos and NFATc1 expression to drive osteoclast differentiation, fusion, and enhanced bone resorption activity (19, 20).

OPG, a soluble decoy receptor secreted by osteoblastic lineage cells, competitively binds RANKL to block RANK activation (21). Therefore, the OPG/RANKL ratio in the local bone microenvironment serves as an important indicator of bone resorption tendency. Regulation of this axis represents a common focus for both anti-resorptive therapies and TCM mechanism research.

### 3.4 Crosstalk between the two pathways in bone coupling

Wnt/β-catenin and OPG/RANKL/RANK pathways are not parallel and isolated. Previous studies demonstrate that Wnt/β-catenin activation can directly upregulate OPG promoter activity and inhibit RANKL expression, thereby suppressing bone resorption while promoting bone formation (16). Conversely, inflammatory cytokines, glucocorticoids, oxidative stress, and AGEs/RAGE can simultaneously upregulate DKK1, SOST, or promote RANKL expression, striking osteogenesis while exacerbating bone resorption (22, 23).

This crosstalk provides the molecular basis for TCM's bidirectional regulation. The “upstream microenvironment dual pathway crosstalk bone coupling reconstruction” framework better accommodates TCM's multi-component, multi-level pharmacological characteristics than linear single-node regulation models (7).

## 4 Pathological subtype differences in osteoporosis

### 4.1 Postmenopausal osteoporosis (PMOP): estrogen deficiency-driven mechanisms

PMOP is characterized by high-turnover bone loss driven by estrogen deficiency. Following estrogen withdrawal, T-cell and osteocyte-derived RANKL increases, the OPG/RANKL ratio decreases, and osteoclast activity rises. Simultaneously, BMSC osteogenic differentiation capacity declines and Wnt signaling activity weakens (24, 25). The resulting bone loss is rapid and primarily affects trabecular bone, explaining the increased fracture risk at vertebral and distal radial sites.

From a TCM intervention perspective, kidney-tonifying and essence-replenishing methods, along with flavonoid constituents possessing phytoestrogen-like characteristics, are particularly suitable for this pathological background. The advantage lies not in simple “estrogen replacement” analogy but in simultaneously correcting both abnormal RANKL elevation and impaired osteogenic capacity (26).

### 4.2 Senile osteoporosis: age-related decline in bone formation and microenvironmental deterioration

Senile osteoporosis usually occurs in older adults of both sexes and is characterized by reduced bone formation, impaired osteoblast function, increased marrow adiposity, osteocyte dysfunction, accumulation of senescent cells, and decreased mechanical loading. Compared with postmenopausal osteoporosis, in which estrogen deficiency frequently induces a high-turnover state with prominent osteoclast activation, senile osteoporosis is often dominated by low bone formation and impaired bone quality (27, 28). At the molecular level, age-related suppression of Wnt/beta-catenin signaling is closely associated with reduced osteogenic differentiation of bone marrow mesenchymal stem cells (BMSCs). Increased expression of Wnt inhibitors, particularly sclerostin (SOST) and Dickkopf-1 (DKK1), may further impair beta-catenin stabilization and downstream osteogenic transcription. In parallel, senescence-associated secretory phenotype (SASP) factors, including inflammatory cytokines and oxidative mediators, may indirectly increase RANKL expression or reduce the OPG/RANKL ratio in the bone microenvironment. However, the OPG/RANKL/RANK axis in senile osteoporosis may be more heterogeneous than in postmenopausal osteoporosis, with some patients showing only mild or variable increases in bone resorption (29). Existing TCM-related studies on senile osteoporosis remain limited. Most available evidence is derived from aging-cell models, D-galactose-induced aging models, naturally aged rodents, or network-pharmacology analyses. Therefore, the therapeutic rationale for senile osteoporosis should emphasize restoration of osteogenic potential, anti-senescence effects, reduction of oxidative stress, improvement of marrow microenvironment, and possible modulation of sclerostin/DKK1-mediated Wnt inhibition. Future studies should use naturally aged animals, include both male and female subjects, and assess clinically relevant outcomes such as bone strength, falls, sarcopenia, and fracture risk.

### 4.3 Secondary osteoporosis: glucocorticoid-induced and inflammatory mechanisms

Glucocorticoid-Induced Osteoporosis (GIOP): The core pathology involves glucocorticoid-induced suppression of osteoblast generation and promotion of osteocyte apoptosis. Early disease may show transiently increased bone resorption, followed by persistent low-turnover bone loss (30, 31). Glucocorticoids upregulate SOST and DKK1, inhibiting canonical Wnt signaling—representing a key molecular basis for GIOP (32).

Diabetic Osteoporosis (DOP): DOP is not simply “diabetes with low bone mass.” Its essence lies in adverse bone microenvironment shaped by hyperglycemia, AGEs/RAGE, chronic low-grade inflammation, oxidative stress, and marrow adiposity (33). Under these conditions, Wnt/β-catenin signaling is suppressed, BMSCs more readily differentiate toward adipogenic lineage, and inflammation-related RANKL elevation further promotes bone resorption (34).

### 4.4 Diabetic osteoporosis: hyperglycemia and advanced glycation end products

Diabetic osteoporosis (DOP) is a complication of diabetes mellitus characterized by deteriorated bone quality and increased fracture risk despite normal or elevated BMD. Both type 1 and type 2 diabetes are associated with increased osteoporosis risk, although the underlying mechanisms may differ (35).

Hyperglycemia and the accumulation of advanced glycation end products (AGEs) are central pathogenic factors in DOP. AGEs can bind to their receptor (RAGE) on osteoblasts and osteoclasts, activating downstream inflammatory signaling pathways and promoting oxidative stress (36). Hyperglycemia may also impair osteoblast function and promote adipogenic differentiation of bone marrow mesenchymal stem cells at the expense of osteogenic differentiation. In addition, diabetes is associated with microvascular complications, neuropathy, and increased fall risk, further contributing to fracture risk (37).

### 4.5 Rationale for focusing on specific osteoporosis subtypes

The present review focuses primarily on postmenopausal osteoporosis, senile osteoporosis, glucocorticoid-induced osteoporosis, and diabetic osteoporosis because these subtypes have relatively abundant experimental evidence linking TCM interventions with Wnt/beta-catenin and OPG/RANKL/RANK signaling. Other clinically important forms of osteoporosis, including male osteoporosis, chronic kidney disease-mineral and bone disorder-related skeletal fragility, rheumatoid arthritis-associated osteoporosis, and cancer therapy-induced bone loss, were not discussed in detail for three reasons. First, their pathophysiology involves additional disease-specific mechanisms, such as androgen deficiency, uremic toxins, parathyroid hormone/fibroblast growth factor 23 imbalance, chronic systemic inflammation, aromatase inhibitor exposure, androgen deprivation therapy, and tumor-bone interactions. Second, TCM studies directly evaluating Wnt/beta-catenin and OPG/RANKL/RANK pathways in these subtypes remain sparse and heterogeneous. Third, combining all subtypes within one review would reduce mechanistic specificity and risk overgeneralization. Nevertheless, these subtypes are clinically important and should be addressed in future focused reviews and experimental studies.

### 4.6 Molecular differences in Wnt/β-catenin and OPG/RANKL/RANK across subtypes

Wnt/β-catenin suppression appears to be a shared feature across PMOP, senile OP, GIOP and DOP, but the dominant upstream drivers differ. In PMOP, reduced Wnt activity is closely linked to estrogen deficiency and immune activation, whereas in senile OP, BMSC senescence and elevated SOST are more prominent. In GIOP, glucocorticoid-induced osteocyte apoptosis with increased SOST and DKK1 leads to sustained β-catenin inhibition; in DOP, AGE/RAGE signalling and chronic inflammation suppress Wnt while shifting BMSC differentiation toward adipogenesis. The OPG/RANKL/RANK axis shows greater heterogeneity: PMOP and DOP exhibit a relatively clear RANKL elevation and decreased OPG/RANKL ratio, while senile OP often shows normal or only mildly altered values, and GIOP may progress from an early resorptive phase to a later low-turnover imbalance. These subtype-specific patterns imply that TCM interventions should be matched to the dominant molecular defect, with RANKL/OPG modulation more relevant for PMOP and inflammation-driven DOP, and restoration of Wnt signalling via SOST/DKK1 inhibition or β-catenin activation more appropriate for senile OP and GIOP. However, because most available studies have pooled subtypes or examined only selected pathway components, these molecular distinctions remain preliminary and require confirmation in subtype-stratified preclinical and clinical investigations (Table 1).

**Table 1:** Molecular characteristics of osteoporosis subtypes and corresponding TCM intervention strategies.

Pathological subtype	Key microenvironment abnormalities	Wnt/β-catenin axis changes	OPG/RANKL/RANK axis changes	TCM intervention focus	Evidence level (highest available)
PMOP	Estrogen deficiency, high bone turnover, immune activation	Decreased osteogenic differentiation capacity, reduced Wnt activity	Elevated RANKL, decreased OPG/RANKL ratio	Kidney-tonifying (Shen-tonifying) and essence-replenishing; flavonoids with phytoestrogen-like properties (e.g., icariin)	Level 2b (small RCTs, *n* < 200); Level 4 (OVX rodent models)
Senile OP	BMSC senescence, marrow adiposity, reduced mechanical loading	Impaired Wnt signaling, increased SOST	Variable, often normal or mildly elevated	Comprehensive microenvironment regulation; anti-senescence strategies; limited specific TCM formulas validated	Level 4–5 (predominantly cellular and senescence-model studies)
GIOP	Glucocorticoid exposure, osteocyte apoptosis, marrow adiposity	Elevated SOST, DKK1; suppressed β-catenin signaling	Early enhanced resorption, later persistent low-turnover imbalance	Rescue Wnt suppression; reduce marrow adiposity; improve microcirculation (e.g., salvianolic acid B, astragaloside IV)	Level 4 (prednisone-treated rodent models)
DOP	AGEs/RAGE, ROS, chronic inflammation, adipogenic bias	Wnt suppression, BMSC adipogenic differentiation shift	Inflammation-related RANKL elevation, secondary enhanced resorption	Antioxidant, anti-inflammatory; block AGE/RAGE; reverse osteogenic/adipogenic imbalance (e.g., morroniside, berberine)	Level 4 (diabetic rodent models)

## 5 Traditional Chinese medicine regulating Wnt/β-catenin and OPG/RANKL/RANK pathways

### 5.1 TCM formulas

Kidney-Tonifying Formulas: Kidney-tonifying represents the core therapeutic principle for osteoporosis in TCM. Systematic reviews indicate that kidney-tonifying (Tonifying-Shen) formulas demonstrate potential benefits in improving bone density, pain, and certain bone metabolism markers, though overall evidence quality remains moderate to low, primarily limited by small sample sizes, inadequate blinding, and short follow-up periods (38). Specifically, the 2020 meta-analysis by Liang et al. (38) included 23 RCTs (*n* = 1,674) but rated only 2 studies as low risk of bias for random sequence generation, and none reported trial registration or sample size calculation. Specifically, the 2020 meta-analysis by Liang et al. (33) included 23 RCTs (*n* = 1,674) but rated only 2 studies as low risk of bias for random sequence generation, and none reported trial registration or sample size calculation.

Zuogui Wan: Recent systems pharmacology research on Zuogui Wan for PMOP suggests that its multi-component synergistic effects may involve Wnt, PI3K/Akt, inflammation, and endocrine-related nodes (39). The value of classical kidney-tonifying formulas lies not only in “promoting osteogenesis” but also in restoring favorable systemic conditions for dual pathway regulation through modulation of inflammation, oxidative stress, hormone deficiency, and marrow microenvironment.

Xianling Gubao (XLGB): XLGB is among the most widely used anti-osteoporosis proprietary medicines in China. Meta-analyses suggest potential improvements in bone density, pain, and certain bone metabolism markers, though evidence for new fracture endpoints remains insufficient, and included studies generally suffer from inadequate randomization and blinding reporting (40). Safety Alert: XLGB-associated drug-induced liver injury (DILI) has been repeatedly highlighted in post-marketing surveillance and causality assessment studies (41, 42). The 2020 screening study by Li et al. (41) identified HLA-B*35:01 and CYP2C9*3 alleles as potential susceptibility biomarkers, suggesting an immune-mediated or idiosyncratic mechanism. The 2024 updated RUCAM assessment may regulate probable causality (score 6–7) in documented cases (37). Hepatotoxicity manifestations include elevated ALT/AST (>3× ULN) within 4–12 weeks of initiation, with cholestatic or mixed patterns. Clinicians should monitor liver function at baseline, 4 weeks, and every 12 weeks thereafter. Given this safety profile, XLGB should not be positioned as an unconditionally “safe natural alternative” to conventional anti-osteoporotics.

Yougui Wan: Yougui Wan is another classical kidney-tonifying formula traditionally used for conditions attributed to kidney-yang deficiency. In the context of osteoporosis, its therapeutic rationale differs partially from Zuogui Wan: whereas Zuogui Wan is more often interpreted as nourishing kidney essence and yin, Yougui Wan is commonly associated with warming kidney yang, strengthening essence, and supporting musculoskeletal function. Recent evidence, indicates that Yougui Wan may be relevant to osteoporosis research. However, because the available evidence appears to remain limited and requires careful verification of clinical and mechanistic endpoints, Yougui Wan should be presented as a promising but not yet definitively validated formula. Future studies should clarify whether its anti-osteoporotic effects involve Wnt/beta-catenin activation, OPG/RANKL/RANK modulation, endocrine regulation, improvement of muscle-bone interaction, or systemic metabolic effects (43).

### 5.2 Proprietary Chinese medicines

In addition to classical formulas, several proprietary Chinese medicines have been developed and marketed for the treatment of osteoporosis. These products are manufactured using standardized processes and quality control measures, which may improve consistency and reproducibility compared with individualized formulas.

Xianling Gubao has been extensively studied in both preclinical and clinical settings. Mechanistic studies have suggested that XLGB may regulate bone metabolism through multiple pathways, including enhancement of osteoblast activity, inhibition of osteoclastogenesis, and modulation of the OPG/RANKL/RANK balance. Clinical studies have reported improvements in BMD and bone turnover markers, although well-designed randomized controlled trials (RCTs) with fracture endpoints are limited.

### 5.3 Active constituents

#### 5.3.1 Flavonoids: icariin as a representative “Pro-osteogenic + anti-osteoclastic” model

Flavonoids represent among the most extensively studied pro-osteogenic active constituents, with icariin being the most representative. Multiple reviews and animal studies demonstrate that icariin promotes Wnt/β-catenin signaling activation, enhances Runx2, ALP, OCN expression, and improves bone microarchitecture and bone density in ovariectomy, disuse, and aging models (44). Simultaneously, icariin reportedly improves the OPG/RANKL ratio and inhibits NF-κB/NFATc1-mediated osteoclast differentiation, presenting a “parallel osteogenic and anti-osteoclastic” bone protection pattern (45). Pharmacokinetic Caveat: Despite robust *in vitro* and rodent efficacy, icariin exhibits extremely poor oral bioavailability in humans (46). Peak plasma concentrations (Cmax) after standard doses (e.g., 60 mg/kg in rats) reach only low nanomolar ranges, whereas *in vitro* osteogenic effects typically require 10–100 *μ*M. This concentration–efficacy disconnect raises fundamental questions about whether observed rodent effects translate via direct skeletal action or through gut microbiota metabolites (e.g., icaritin, desmethylicaritin), which may serve as active proxies. Future formulation development (e.g., lipid nanoparticles, bone-targeted delivery) is urgently needed to bridge this PK–PD gap.

#### 5.3.2 Saponins: astragaloside IV and metabolic microenvironment remodeling

Astragaloside IV has garnered attention for osteogenic differentiation and metabolic regulation. Studies indicate that astragaloside IV promotes osteoblast differentiation through modulation of the GSK-3β/β-catenin axis (47). Beyond osteogenesis, astragaloside IV may indirectly create conditions for Wnt signaling recovery through antioxidant effects, improved metabolic stress, and inflammatory microenvironment modulation (48, 49). Oral bioavailability of astragaloside IV is similarly limited (absolute bioavailability ∼2.2% in rats), with rapid systemic clearance (t₁/₂ 34–131.6 min). Encapsulation strategies, such as tetracycline-modified mPEG-PLGA micelles, have shown preliminary success in improving bone accumulation and anti-osteoporotic efficacy in rodent models (50).

#### 5.3.3 Phenolic acids and quinones: salvianolic acid B and tanshinone IIA

Tanshinone IIA has been demonstrated in animal experiments to significantly reduce OSCAR and CTX-1 expression, inhibit RANKL-induced c-fos and NFATc1 expression, reduce TRAP-positive multinucleated osteoclast formation, and suppress osteoclastogenesis (51). It covalently binds LDHC, inhibiting its enzymatic activity, reducing ROS accumulation, and modulating antioxidant and anti-inflammatory activity (52). Tanshinone IIA is a lipophilic diterpene with moderate oral absorption but high plasma protein binding (>90%), potentially limiting free drug availability to bone tissue.

Salvianolic acid B directly increases RUNX2 and OCN expression, activates MEK-ERK pathway through p-ERK elevation, and increases TAZ expression to regulate osteoblast differentiation (53). In zebrafish experiments, salvianolic acid B stimulated bone formation by counteracting oxidative stress and increasing osteoblast-specific gene expression (54). Salvianolic acid B SalB accelerates osteoporotic fracture healing and improves the mechanical properties of the fracture site by regulating the lncRNA-MALAT1/miR-155-5p/HIF-1*α* signaling axis, while promoting osteogenic differentiation and type H vessel formation (55).

#### 5.3.4 Alkaloids: Berberine's anti-osteoclastic and anti-inflammatory effects

Berberine suggests relatively clear mechanisms in osteoclast lineage. Studies show berberine inhibits RANKL-induced NF-κB and Akt activation, reducing osteoclast formation and survival (56). From a pathway perspective, it more prominently suggests bone resorption side inhibition, potentially producing indirect bone metabolism benefits through improved inflammatory and metabolic environments (57). Critical PK/PD Gap: Berberine is a well-characterized P-glycoprotein substrate with oral bioavailability <1% in humans. Effective *in vitro* concentrations (5–50 *μ*M) are rarely achievable systemically. Paradoxically, berberine modulates gut microbiota composition (e.g., increasing Akkermansia muciniphila) and exerts anti-inflammatory effects via the intestinal–bone axis, suggesting that its anti-osteoporotic action may be largely gut-mediated rather than direct skeletal targeting (57). This challenges simplistic “target-binding” narratives and underscores the need for gut–bone axis research in TCM pharmacology.

Gallic acid: Gallic acid is a naturally occurring phenolic acid present in various medicinal plants and foods. Emerging evidence suggests that gallic acid may exert bone-protective effects through antioxidant, anti-inflammatory, and anti-osteoclastogenic mechanisms. In osteoporosis-related models, gallic acid has been reported to reduce oxidative stress, suppress inflammatory mediators, inhibit RANKL-induced osteoclast differentiation, and potentially support osteoblast differentiation under adverse microenvironmental conditions. These effects are mechanistically relevant to diabetic osteoporosis and inflammation-associated bone loss, in which reactive oxygen species (ROS), inflammatory cytokines, and RANKL-mediated osteoclastogenesis contribute to impaired bone coupling. However, current evidence is still mainly preclinical, and human pharmacokinetic data, bone-tissue exposure, optimal dose, long-term safety, and fracture-related efficacy remain insufficiently characterized. Therefore, gallic acid should be considered a promising experimental constituent rather than an established anti-osteoporotic therapy (58) (Table 2).

**Table 2:** Comparative summary of representative TCM interventions targeting Wnt/β-catenin and OPG/RANKL/RANK signaling in osteoporosis.

Intervention	Category	Main proposed targets	Relevant subtype/model	Pharmacokinetic or safety limitation	Evidence level
Icariin (59, 60)	Flavonoid	May activate Wnt/β-catenin; may increase Runx2, ALP, OCN; may improve OPG/RANKL ratio and inhibit NF-κB/NFATc1-mediated osteoclastogenesis	PMOP, OVX models, aging/disuse models	Poor oral bioavailability; uncertain human bone exposure; possible metabolite-mediated effects	Mainly Level 4–5; limited clinical evidence in formulas
Astragaloside IV (61–63)	Saponin	May regulate Wnt signal; Lep, Ptgs2, Gfap, Igfbp2, Il22ra2, Wnt10a, and Wnt1; antioxidant and anti-inflammatory effects	PMOP, OVX, metabolic bone-loss models	Low oral bioavailability; rapid clearance; limited human skeletal PK data	Mainly Level 4–5
Tanshinone IIA (64–66)	Diterpene quinone	May regulate ERK1/2 -CREB, AKT, PPAR signaling pathway signaling; antioxidant and anti-inflammatory effects	Inflammatory osteoporosis, GIOP-related models	High protein binding; uncertain free bone concentration	Mainly Level 4–5
Salvianolic acid B (53–55)	Phenolic acid	May promote lncRNA-MALAT1/miR-155-5p/HIF-1*α*; may activate ERK/TAZ-related osteogenesis; antioxidant effects	GIOP-related models	Limited permeability and systemic exposure; limited human target-engagement evidence	Mainly Level 4
Berberine (56, 57)	Alkaloid	May regulate oxidative stress, NF-kappa B and Akt pathways, and RANKL	DOP-related models	Low oral bioavailability; potential CYP/P-gp-mediated interactions	Mainly Level 4–5
Morroniside (67–69)	Iridoid glycoside	May improve NF-κB and MAPK signaling and Osteoblast and Osteoclast Differentiation	OVX, DOP models	Limited human PK and safety data	Mainly Level 4–5
Gallic acid (58)	Phenolic acid	May regulate Akt, ERK, and JNK pathways and osteoclastogenesis	OVX models	Limited skeletal exposure and clinical data	Mainly Level 4–5
Xianling Gubao (40–42)	Proprietary Chinese medicine	May regulate bone formation/resorption markers and OPG/RANKL balance	PMOP and primary osteoporosis	Reported hepatotoxicity; quality control and herb–drug interaction concerns	Small clinical studies/meta-analyses; fracture evidence insufficient
Zuogui Wan (39, 70, 71)	Classical formula	May involve Wnt/β-catenin signaling, PI3K/Akt, endocrine and inflammatory regulation	PMOP and kidney-deficiency pattern-related OP	Formula complexity; active components and exposure unclear	Mainly network pharmacology, animal, and limited clinical evidence
Yougui Wan (43, 72)	Classical formula	Potential kidney-yang-tonifying effect; CD4, IL-17A, RANKL, and ROR*γ*t	OP with kidney-yang-deficiency pattern; evidence emerging	Requires verification of active constituents, PK, and clinical endpoints	Emerging evidence
Acupuncture/moxibustion (72–74)	Non-pharmacological TCM	Possible neuroendocrine, inflammatory, circulation, and pain-modulating effects; direct Wnt/OPG evidence limited	OP-related pain/function; adjunctive care	Heterogeneous protocols; limited fracture and pathway-biomarker data	Clinical and mechanistic evidence heterogeneous

### 5.4 Acupuncture/moxibustion (non-pharmacological therapies)

Electroacupuncture has shown preliminary regulatory effects on Wnt/β-catenin and OPG/RANKL in ovariectomized rodent models (73, 74). However, the evidence base is limited to small animal studies (Level 4) with significant methodological heterogeneity. High-quality clinical evidence remains absent (75). Acupuncture and moxibustion are non-pharmacological TCM interventions that may influence pain, physical function, neuroendocrine regulation, local circulation, inflammation, and possibly bone metabolism. However, the current evidence linking acupuncture or moxibustion specifically to Wnt/beta-catenin or OPG/RANKL/RANK regulation in osteoporosis remains limited compared with herbal compounds. It is important to distinguish insufficient molecular-pathway evidence from clinical ineffectiveness. A lack of direct evidence on Wnt/beta-catenin or OPG/RANKL/RANK modulation does not mean that acupuncture or moxibustion lacks clinical value. Rather, it indicates that the mechanistic basis remains underexplored. Future studies should combine clinical outcomes with mechanistic endpoints, including bone-turnover markers, inflammatory cytokines, OPG/RANKL ratio, Wnt inhibitors such as SOST and DKK1, and imaging-based bone-density or microarchitecture parameters. Until such evidence is available, acupuncture and moxibustion should be discussed separately from pharmacological TCM interventions and interpreted cautiously.

### 5.5 Subtype-specific TCM interventions

PMOP: The focus is on compensating for estrogen deficiency-induced high-turnover bone loss. Kidney-tonifying and essence-replenishing methods, along with flavonoid constituents possessing phytoestrogen-like characteristics, are particularly suitable. The advantage lies not in simple “estrogen replacement” analogy but in simultaneously correcting both abnormal RANKL elevation and impaired osteogenic capacity (26). Mechanistically, these interventions may partially enhance Wnt/beta-catenin-related osteogenesis and improve the OPG/RANKL ratio, but clinical fracture evidence remains insufficient.

For senile osteoporosis, the therapeutic focus should shift toward improving osteoblast dysfunction, cellular senescence, marrow adiposity, oxidative stress, and muscle-bone interaction. TCM approaches may include formulas aimed at tonifying kidney, strengthening spleen, replenishing qi, activating blood, and improving systemic microenvironment. However, validated formula-specific evidence for senile osteoporosis is still limited, and more studies using naturally aged models are needed.

GIOP: Salvianolic acid B's promotion of osteogenesis and marrow angiogenesis in prednisone-related models suggests unique value for kidney-tonifying and blood-activating approaches in GIOP (47). The main pathological mechanisms include suppression of osteoblastogenesis, osteocyte apoptosis, increased SOST and DKK1 expression, and early osteoclast activation followed by low-turnover bone loss. TCM interventions with potential relevance include salvianolic acid B, astragaloside IV, tanshinone IIA, and blood-activating or qi-tonifying formulas. The mechanistic focus should be rescue of Wnt suppression, reduction of oxidative stress, and improvement of microcirculation rather than simple anti-resorption.

DOP: The key microenvironmental abnormalities include hyperglycemia, advanced glycation end products (AGEs), oxidative stress, chronic low-grade inflammation, and adipogenic differentiation of BMSCs. Berberine, morroniside, gallic acid, and antioxidant or anti-inflammatory formulas may be particularly relevant. Their potential mechanisms include modulation of AGE/RAGE signaling, reduction of reactive oxygen species (ROS), improvement of osteogenic/adipogenic balance, and suppression of inflammation-related RANKL expression. Nevertheless, evidence remains mainly preclinical, and clinical validation is required.

## 6 Bone coupling as an integrative framework

### 6.1 Osteoclast-derived coupling factors

Bone coupling refers to the tight spatiotemporal coordination between bone resorption and formation during bone remodeling. Beyond the classical OPG/RANKL/RANK axis, osteoclasts secrete multiple coupling factors that directly influence osteoblast activity:

Sphingosine-1-phosphate (S1P): Released during bone resorption, S1P recruits osteoblast precursors to resorption lacunae (76).

Platelet-derived growth factor-BB (PDGF-BB): Secreted by pre-osteoclasts, promotes osteoblast migration and differentiation (77).

Connective tissue growth factor (CTGF): Facilitates osteoblast function and bone matrix deposition (78).

Bone morphogenetic proteins (BMPs): Released from bone matrix during resorption, stimulate osteogenesis (79).

Transforming growth factor-β (TGF-β): Liberated from demineralized matrix, regulates both osteoclast and osteoblast activity (80).

### 6.2 TCM modulation of coupling signals

TCM's multi-target characteristics may confer unique advantages in modulating bone coupling. Icariin not only affects Wnt/β-catenin and OPG/RANKL/RANK pathways but also modulates S1P signaling and PDGF-BB expression (81). Salvianolic acid B's promotion of marrow angiogenesis may improve the vascular niche required for coupling factor delivery (53). These effects cannot be fully explained by single-pathway models and require integrative bone coupling frameworks for comprehensive understanding. However, direct evidence linking specific TCM constituents to coupling factor modulation remains sparse. Most assertions (e.g., icariin → PDGF-BB) derive from indirect inference rather than lineage-tracing or conditional knockout validation. We classify these as “hypothesis-generating” (Level 5) rather than established mechanisms.

### 6.3 Resorption-independent anabolic effects of pre-osteoclasts

Recent studies have revealed that pre-osteoclasts (osteoclast precursors before full differentiation) can exert bone anabolic effects independent of bone resorption. These cells secrete PDGF-BB and other factors that promote osteogenesis without requiring actual bone resorption (57). This discovery challenges the traditional view that osteoclasts only function in bone resorption and suggests new therapeutic targets. TCM constituents that modulate osteoclast differentiation at early stages may potentially harness these anabolic effects while suppressing mature osteoclast-mediated resorption (82).

## 7 Discussion

### 7.1 TCM multi-target, multi-pathway synergistic advantages vs. western medicine single-target limitations

Western anti-osteoporotic drugs have evolved from single-node inhibitors (e.g., bisphosphonates targeting farnesyl pyrophosphate synthase) to dual-action biologics such as romosozumab, which neutralizes sclerostin to simultaneously enhance bone formation and indirectly suppress resorption (83). Even “single-target” agents like denosumab (anti-RANKL) produce complex downstream network effects. These therapies, while effective, carry specific risks: atypical femoral fractures (bisphosphonates), rebound vertebral fractures after denosumab discontinuation (84), and rodent osteosarcoma signals (teriparatide) (85, 86).

TCM bioactive constituents (e.g., icariin, berberine, tanshinone IIA) frequently exhibit polypharmacology, modulating multiple nodes within Wnt/β-catenin and OPG/RANKL/RANK pathways concurrently. This “soft regulation” may theoretically produce more physiologically harmonious bone remodeling by fine-tuning microenvironmental signals rather than forcing a unidirectional shift. However, polypharmacology also implies lower target specificity, higher risk of off-target interactions (e.g., CYP450 inhibition by berberine), and greater batch-to-batch variability due to herbal source heterogeneity. The challenge lies not in celebrating multi-target effects *per se*, but in deconvolving network-level observations into verifiable, reproducible target–dose–response relationships (87). Network pharmacology predictions (e.g., TCMSP database) remain largely computational; experimental validation via gene knockout, lineage tracing, and target engagement biomarkers is urgently needed to bridge the prediction–verification gap (88).

### 7.2 Current research limitations

#### 7.2.1 Pharmacokinetic and exposure-related limitations

Although many TCM-derived compounds show osteogenic or anti-osteoclastogenic effects *in vitro*, their translational feasibility depends on whether effective concentrations can be achieved at skeletal sites *in vivo*. A recurring limitation is the discrepancy between micromolar concentrations used in cell experiments and much lower systemic exposure after oral administration. For example, icariin, berberine, and several phenolic compounds have low oral bioavailability due to poor solubility, limited intestinal permeability, efflux transport, extensive metabolism, or rapid clearance (89, 90).

Importantly, low plasma concentration does not automatically indicate clinical inactivity, because active metabolites, gut microbiota-mediated biotransformation, tissue accumulation, immunomodulatory effects, or indirect endocrine/metabolic regulation may contribute to biological activity (91, 92). Nevertheless, most studies have not measured free drug concentration, active metabolite exposure, bone marrow distribution, or target engagement in bone tissue. The feasibility of achieving *in vitro* effective concentrations in human bone therefore remains uncertain.

Rodent doses should not be directly extrapolated to humans without body-surface-area conversion, exposure comparison, and pharmacodynamic validation. Future studies should integrate pharmacokinetics, pharmacodynamics, metabolomics, microbiome analysis, and bone-targeted delivery strategies. Nanoparticles, liposomes, phospholipid complexes, and bone-affinity carriers may improve skeletal delivery, but these approaches require rigorous safety evaluation before clinical translation.

#### 7.2.2 Safety, herb-drug interactions, and quality-control issues

Safety evaluation is essential because “natural” does not mean risk-free. Xianling Gubao-associated drug-induced liver injury has been reported in post-marketing surveillance and causality-assessment studies, suggesting that liver function should be monitored before and during treatment, especially in patients with pre-existing liver disease, polypharmacy, alcohol use, or advanced age (42).

Beyond hepatotoxicity, several additional safety domains require attention. First, herb-drug interactions may occur through cytochrome P450 enzymes, P-glycoprotein, organic anion transporters, or additive pharmacodynamic effects. This is particularly relevant because patients with osteoporosis are often older adults receiving bisphosphonates, denosumab, vitamin D, calcium, anticoagulants, antidiabetic drugs, antihypertensives, statins, glucocorticoids, or immunosuppressants. Second, nephrotoxicity should be considered in formulas containing potentially nephrotoxic herbs or in patients with chronic kidney disease. Third, reproductive and endocrine safety should be evaluated for phytoestrogen-like compounds, especially in hormone-sensitive conditions. Fourth, long-term toxicity data are insufficient for many isolated constituents and compound formulas used chronically for osteoporosis prevention. Fifth, quality control remains a major translational challenge because herbal preparations may vary in botanical origin, harvest season, processing method, extraction procedure, active-ingredient content, contaminants, pesticide residues, heavy metals, and adulterants.

#### 7.2.3 Other limitations

Sample Size and Study Quality: Most TCM osteoporosis studies remain small-scale cellular and animal experiments. Clinical trials often suffer from inadequate randomization, lack of blinding, short follow-up periods, and absence of hard endpoints (fracture incidence) (33). As of 2025, no TCM intervention for osteoporosis has completed a Phase III RCT with fracture incidence as the primary endpoint registered on ClinicalTrials.gov.

Fragmented Mechanism Research: Studies often focus on single constituents or single pathways, failing to capture the network pharmacology characteristics of TCM formulas. The gap between network prediction and experimental verification remains substantial (68).

Lack of High-quality RCTs: Existing meta-analyses are limited by low-quality original studies. Large-scale, multi-center, randomized, double-blind, placebo-controlled trials with fracture endpoints are urgently needed (93).

Safety Concerns: The assumption that “natural equals safe” is misleading. XLGB-associated liver injury exemplifies the necessity for rigorous safety monitoring of TCM proprietary medicines (36, 37). Additional safety concerns include: (1) herb–drug interactions (e.g., St. John's wort–like CYP induction by some TCMs, though evidence for osteoporosis formulas is sparse); (2) adulteration with undeclared synthetic drugs (e.g., corticosteroids, NSAIDs) in unregulated proprietary medicines; (3) heavy metal and aflatoxin contamination in poorly sourced herbal materials. These risks demand pharmacovigilance systems equivalent to those for synthetic drugs.

### 7.3 Future directions

Future research should address five priorities. First, study selection and reporting should be more transparent, with prospective protocols, predefined outcomes, and PRISMA-style flow diagrams even for structured narrative reviews. Second, preclinical studies should improve methodological rigor through randomization, allocation concealment, blinded outcome assessment, sample-size calculation, and reporting according to ARRIVE/SYRCLE recommendations. Third, pharmacokinetic-pharmacodynamic studies should determine whether active compounds or metabolites reach bone tissue at biologically relevant concentrations and whether pathway-level target engagement occurs *in vivo*. Fourth, clinical trials should use standardized, quality-controlled TCM products and include clinically meaningful endpoints, particularly incident vertebral and non-vertebral fractures, in addition to BMD and bone-turnover markers. Fifth, subtype-specific trials should be designed for PMOP, senile osteoporosis, GIOP, and DOP because these conditions differ substantially in Wnt suppression, osteoclast activation, metabolic inflammation, and bone-turnover status.

Network Pharmacology and Systems Biology: Integration of UPLC-MS, metabolomics, proteomics, and transcriptomics can elucidate multi-component synergistic mechanisms (94, 95). However, computational predictions must be validated through cell-specific interventions, gene knockout/knockdown, and lineage tracing (96).

Precision Medicine: Stratified research according to pathological subtypes (PMOP, GIOP, DOP) rather than simple mixing. Different subtypes require different TCM intervention strategies targeting their specific microenvironmental abnormalities.

Bone Coupling-Targeted Strategies: Development of TCM formulations that specifically modulate coupling factors (S1P, PDGF-BB, CTGF) may achieve more physiologically harmonious bone remodeling than simple osteoblast stimulation or osteoclast inhibition.

Drug Delivery Systems: Bone-targeted nanoparticle delivery systems, sustained-release formulations, and novel administration platforms can increase local concentration and bioavailability of active constituents in bone tissue while reducing systemic exposure. For example, bisphosphonate-conjugated nanoparticles or hydroxyapatite-coated liposomes could concentrate icariin or berberine at resorption lacunae, bypassing systemic PK limitations (97–99).

Standardization and Quality Control: Establishment of standardized preparations, batch consistency, and pharmacokinetic-pharmacodynamic relationships to bridge the gap between basic research and clinical practice. This includes quantification of marker compounds by UPLC-MS/MS, heavy metal/aflatoxin screening, and stability testing under ICH Q1A conditions (100–102).

### 7.4 Pharmacokinetic and translational barriers

A critical but underappreciated limitation in TCM osteoporosis research is the disconnect between *in vitro* target engagement and *in vivo* bone exposure. As summarized in Table 2, flagship constituents (icariin, astragaloside IV, berberine) exhibit oral bioavailability <5% in humans, with systemic concentrations orders of magnitude below *in vitro* EC50 values. This raises three possibilities: (1) bone tissue achieves higher local concentrations than plasma (unproven for most constituents); (2) gut microbiota metabolites (e.g., icaritin from icariin) are the true active entities; or (3) observed rodent efficacy reflects species-specific PK or high-dose artifacts. Without validated bone-targeted delivery systems or human target-engagement biomarkers (e.g., β-catenin phosphorylation status in circulating osteoprogenitors), claims of “Wnt activation” or “RANKL inhibition” by oral TCM remain pharmacologically speculative. Future research must integrate microdialysis, PET imaging with radiolabeled constituents, and bone-specific nanoparticle carriers to resolve these uncertainties (103).

### 7.5 Clinical translation and guideline relevance

Current clinical evidence for TCM in osteoporosis is weaker than that for established anti-osteoporotic drugs such as bisphosphonates, denosumab, teriparatide, abaloparatide, and romosozumab. Some randomized or quasi-randomized studies and meta-analyses suggest that kidney-tonifying formulas or proprietary Chinese medicines may improve bone mineral density (BMD), pain scores, or bone-turnover markers. However, most trials have small sample sizes, short follow-up periods, unclear randomization, inadequate allocation concealment, insufficient blinding, heterogeneous interventions, and limited adverse-event reporting.

Most importantly, fracture reduction—the most clinically meaningful endpoint in osteoporosis—has rarely been evaluated as a primary outcome in adequately powered randomized controlled trials of TCM interventions. Therefore, TCM should not currently be presented as a replacement for guideline-recommended anti-fracture pharmacotherapy in high-risk patients. Instead, its potential role may be considered as adjunctive or complementary, provided that product quality, safety monitoring, drug interactions, and patient-specific risk factors are carefully addressed.

## 8 Conclusion

Wnt/β-catenin and OPG/RANKL/RANK pathways constitute the two principal axes of bone coupling regulation and represent the most explanatory molecular framework for understanding TCM's anti-osteoporotic effects. Current research supports that TCM monomers, compound formulas, and proprietary medicines exert comprehensive bone protection through promoting osteogenesis, inhibiting osteoclastogenesis, and reshaping bone microenvironment. However, this effect is not simple linear stimulation or inhibition of dual pathways but rather resembles flexible regulation of the “microenvironment signaling network cell fate” system as a whole (Figure 2). Nevertheless, the evidence base remains heavily weighted toward preclinical studies (Levels 4–5), with significant pharmacokinetic barriers (low oral bioavailability, unclear bone exposure) and safety concerns (XLGB hepatotoxicity, herb–drug interaction potential) limiting current clinical translation.

**Figure 2:**
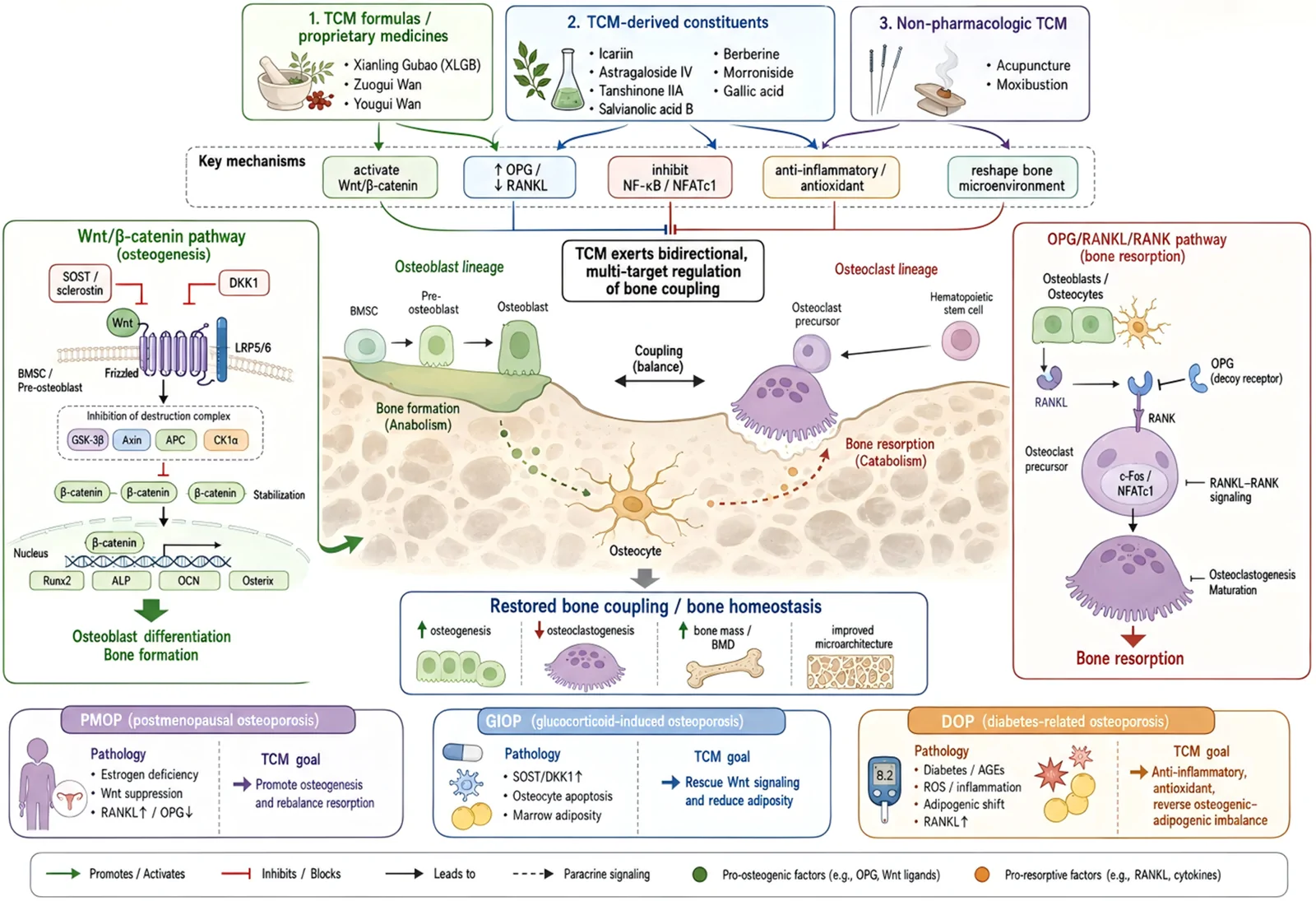
Schematic representation of TCM-mediated regulation of bone coupling through Wnt/beta-catenin and OPG/RANKL/RANK pathways.

Pathological subtype differences (PMOP, GIOP, DOP) determine distinct therapeutic focuses: PMOP emphasizes compensating for estrogen deficiency-induced high RANKL states; GIOP emphasizes rescuing suppressed osteoblast axis; DOP requires simultaneous management of metabolic inflammation and adipogenic bias. Future research should integrate multi-omics, spatial biology, standardized efficacy evaluation, and randomized controlled trials to advance TCM osteoporosis research from “correlation description” to “causal verification” and clinical translation. Priority should be given to: (1) human target-engagement studies to validate pathway modulation at clinically achievable exposures; (2) bone-targeted drug delivery to overcome PK limitations; (3) rigorous hepatovigilance and pharmacovigilance protocols for proprietary medicines; and (4) at least one adequately powered RCT (*n* > 1,000) with fracture endpoints to establish efficacy beyond bone mineral density surrogates.
